# Pathogenicity and Volatile Nematicidal Metabolites from *Duddingtonia flagrans* against *Meloidogyne incognita*

**DOI:** 10.3390/microorganisms9112268

**Published:** 2021-10-31

**Authors:** Xiaoyu Mei, Xin Wang, Guohong Li

**Affiliations:** State Key Laboratory for Conservation and Utilization of Bio-Resources in Yunnan, and Key Laboratory for Southwest Microbial Diversity of the Ministry of Education, Yunnan University, Kunming 650032, China; 15808859550@139.com

**Keywords:** nematode-trapping fungi, *Duddingtonia flagrans*, *Meloidogyne incognita*, VOCs, cyclohexanamine, egg hatching

## Abstract

Plant parasitic nematodes, especially parasitic root-knot nematodes, are one of the most destructive plant pathogens worldwide. The control of plant root-knot nematodes is extremely challenging. *Duddingtonia flagrans* is a type of nematode-trapping fungi (NTF), which produces three-dimensional adhesive networks to trap nematodes. In this study, the pathogenicity and volatile organic compounds (VOCs) of the NTF *D. flagrans* against the plant root-knot nematode, *Meloidogyne incognita*, were investigated. The predatory process of *D. flagrans* trapping *M. incognita* was observed using scanning electron microscopy. Gas chromatography-mass spectrometry analysis of the VOCs from *D. flagrans* led to the identification of 52 metabolites, of which 11 main compounds were tested individually for their activity against *M. incognita*. Three compounds, cyclohexanamine, cyclohexanone, and cyclohexanol, were toxic to *M. incognita*. Furthermore, these three VOCs inhibited egg hatching of *M. incognita*. Cyclohexanamine showed the highest nematicidal activity, which can cause 97.93% mortality of *M. incognita* at 8.71 µM within 12 h. The number of hatched juveniles per egg mass after 3 days was just 8.44 when treated with 26.14 µM cyclohexanamine. This study is the first to demonstrate the nematicidal activity of VOCs produced by *D. flagrans* against *M. incognita*, which indicates that *D. flagrans* has the potential to biocontrol plant root-knot nematodes.

## 1. Introduction

Plant parasitic nematodes infect a variety of crops and cause severe damage, which leads to annual economic losses estimated at $173 billion [1]. Among plant parasitic nematodes, root-knot nematodes (*Meloidogyne* spp.) cause the greatest losses in agriculture [2]. Chemical nematicides have been used to control them for a long time, which has led to resistance in nematodes and caused environmental problems. Biological control is an effective and popular method for reducing the parasite population because it does not have an impact on the environment. Nematophagous fungi, natural enemies of plant parasitic nematodes, have been suggested as promising resources for the biological control of nematodes. They can exist in diverse environments, and some of them can switch from being saprotrophic to predatory when nematodes are present [3]. Nematophagous fungi include four major groups; namely, trapping, endoparasitic, opportunistic, and toxic fungi [4,5,6]. Among them, nematode-trapping fungi (NTF) can produce diverse trap devices containing adhesive network, branches, knobs and constricting or non-constricting rings to capture nematodes [7].

*Duddingtonia flagrans* is a representative species of NTF and belongs to the family Orbiliaceae of Ascomycota. It forms three-dimensional adhesive networks that trap the nematodes. *Duddingtonia flagrans* is considered as a promising fungal species for the biological control of animal endoparasites as several reports showed its effectiveness in the control of gastrointestinal parasites [8,9,10,11]. *Duddingtonia flagrans* has attractive potential as a biocontrol agent, because it can easily produce a large quantity of chlamydospores that are appropriate for dissemination in the environment [3]. However, to date, there have been few reports on the use of this fungus to control plant-parasitic nematodes. A *D. flagrans* isolate was assessed for its trapping efficiency against the root-knot nematode *Meloidogyne* spp. [12]. Subsequently, Monteiro et al. proved that *D. flagrans* reduced 72.7% of juveniles of *Meloidogyne javanica* at a concentration of 8201 chlamydospores per gram of soil [13], which illustrates the ability to control plant parasitic nematodes of the species. Hence, *D. flagrans* has the potential to be developed as a biological control agent against plant parasitic nematodes. However, there is no information about the predatory process of this fungal species on the plant’s parasitic nematode. In addition to controlling nematodes, *D. flagrans* can promote growth and phosphorus use efficiency in tomato plants, which increases its potential uses [14]. 

Many microorganisms and their metabolites have shown great potential for the control of nematodes. Volatile organic compounds (VOCs) are natural small volatile substances that are generally less toxic and have great potential for application as biological control agents [15]. Nematicidal VOCs produced by microorganisms are thus considered promising molecules to develop nematicides. Many nematicidal VOCs have been identified in fungi. 2-Methylbutyl acetate, 3-methylbutyl acetate, ethyl acetate, and 2-methylpropyl acetate are the main VOCs produced by *Fusarium oxysporum*, which are active against plant parasitic nematodes [16]. Liarzi, et al. found that the fungus *Daldinia* cf. *concentrica* produces nematicidal VOCs that can control the root-knot nematode *M. javanica*, both in vitro and in greenhouse experiments, and demonstrated that 4-heptanone was the major active component [17]. Li, et al. detected the volatile substances released by *Annulohypoxylon* sp. FPYF3050; among them being 1,8-cineole, which showed strong activity against the pine wood nematode *Bursaphelenchus xylophilus* [18]. To date, there are many nematicidal metabolites reported from NTF [19], but few about VOCs. In *D. flagrans*, three metabolites, namely, flagranones A, B, and C, were found, and these compounds displayed antimicrobial activity [20]. 

In the present study, the predatory process of *D. flagrans* against the plant parasitic nematode *M. incognita* was observed using scanning electron microscopy (SEM). Based on the analysis of gas chromatography-mass spectrometry (GC-MS) and confirmation with commercial compounds, three compounds (cyclohexanamine, cyclohexanone, and cyclohexanol) were toxic to both juveniles and eggs of *M. incognita*. This implies that the NTF *D. flagrans* can infect nematodes by shaping the trap device and producing active metabolites.

## 2. Materials and Methods

### 2.1. Materials and Normal Culture

*D. flagrans* (CBS565.50) was deposited in the culture collection of the Key Laboratory for Conservation and Utilization of Bio-resource, and Key Laboratory for Microbial Resources of the Ministry of Education, Yunnan University. The strain was initially maintained on potato dextrose agar (PDA, 200 g potato, 20 g dextrose, 20 g agar, 1 L water, pH 7.0) slants at 4 °C. The strain was transferred to PDA plates at 28 °C for 7 days and then inoculated into 250 mL triangular flasks, each containing corn medium (70 g corn kernels and 70 mL water in a 250 mL flask). In the solid medium, the strain was cultured at 28 °C for 20 days. The cultures (5 g) were taken out and put into another sterilized 50 mL flask, and 5 mL ddH_2_O were added to rinse chlamydospores.

In the volatile experiment, the strain cultured on PDA medium was transferred into a 250 mL flask containing 100 mL of yeast extract peptone dextrose medium (YPD, 20 g peptone, 10 g yeast extract, 20 g dextrose, 1 L water). The liquid cultures were incubated in a rotary shaker (180 rpm) at 28 °C for 5 days as a seed. Then, 2 mL of seed was added to a 250 mL flask containing 100 mL of YPD medium and incubated at 180 rpm at 28 °C for 14 days. Fungal cultures were used in the subsequent experiments.

*M. incognita* egg masses were collected from the roots of infested tomato plants. The method for isolating nematodes from roots refers to reference [21]. The egg masses were incubated at room temperature to obtain juveniles that were collected and prepared in distilled water as a suspension to use.

### 2.2. Observation of Trap Formation and Predatory Process Using Scanning Electron Microscopy

The trap formation and pathogenicity of *D. flagrans* against *M. incognita* were observed using SEM. Dialysis membrane discs (3 cm in diameter) were placed on the surface of water agar medium (WA, 2% agar–water) in 3 cm diameter Petri dishes. Two microliters of chlamydospores (approximately 150–200) were covered on the surface of the WA medium and evenly distributed on the surface of the dialysis membrane using a spreader. These Petri dishes were then incubated at 28 °C. After four days, Petri dishes were removed from the incubator and suspension containing approximately 200-300 J2s of *M. incognita* was added to the *D. flagrans*. These Petri dishes were then incubated continuously at 28 °C. Pieces of dialysis membranes were collected at 12, 24, and 48 h after inoculation with *M. incognita* and cut with a scalpel. The samples were prepared based on the method described by Wan et al., 2021 [22] and observed using a scanning electron microscope. 

### 2.3. Gas Chromatography–Mass Spectrometry Assay 

The fermentation broth of *D. flagrans* cultured on YPD medium was extracted with cyclohexane, dehydrated using anhydrous sodium sulfate, and then quickly concentrated using a rotary evaporator at low temperature and negative pressure. The VOC components of the samples were analyzed by GC-MS (7890A-5975C GC-MS system, Agilent Technologies, Santa Clara, CA, USA). The VOCs were identified from a database comparison of the mass spectrum of the substance with data banks (NIST) [23].

All commercial compounds were purchased from Macklin (Shanghai, China) with a purity of ≥98%. The following chemicals were used: cyclohexanol (≥98.5%), cyclohexanone (98%), cyclohexanamine (98%), heptane (≥98%), 2-pyrrolidinone (99.5%), benzeneethanol (91%), tetradecane (98%), 1-dodecanol (95%), cyclododecane (99%), 9-octadecenoic acid (99%) and tetracosane (99%).

### 2.4. Assays of Nematicidal Activity

#### 2.4.1. Nematicidal Activity of the Broth’s VOCs and Compounds 

Broth (200 µL) cultured in YPD medium, or each commercial VOC at the different doses was added to one well in the center of a 96-well plate respectively; 100 µL of nematode suspension in ddH_2_O (approximately 100–200 worms) were added to the eight wells adjacent to the test sample [24]. The YPD medium (200 µL) was used as a control for the culture filtrate (200 µL) of *D. flagrans*. In the experiment with commercial VOCs, no control was used. The plates were immediately wrapped in Parafilm. All plates were incubated at 25 °C for 6, 12, and 24 h. Mobile and immobile juveniles were counted under a microscope. *Meloidogyne incognita* was considered dead when no movement was observed after it was touched with a needle. The experiment was repeated three times.

The corrected mortality of *M. incognita* was calculated using Schneider–Corelli’s formula [25]. The data in Figure 3A performed a Student *t* test on the control and broth at each time point. Different letters indicate that there is a significant difference between the control and the broth at this time point when *p* < 0.05. The experimental data were averaged and analyzed by SPSS 20. Comparison between groups was analyzed through single-factor ANOVA. LSD was used in post-hoc test.

#### 2.4.2. Inhibition of Egg Hatching Activity by VOCs

The broth and VOCs were tested for the inhibition of the egg hatching activity of *M. incognita*. Egg masses were collected from the infested roots. The experiments were performed in 96-well plates. Broth and each VOC (the concentration of each compound was varied and determined according to its activity result) were added to one well in the center of a 96-well plate independently. An egg mass was placed in distilled water and added to one well adjacent to the sample (altogether eight wells), respectively [26]. The plates were incubated at 25 °C. The numbers of hatched juveniles were counted under a microscope after 1, 2, and 3 days. Each treatment was performed in triplicate and data analysis was the same as above. 

## 3. Results

### 3.1. Trap Formation and Pathogenicity Process of D. flagrans against M. incognita

**Figure 1:**
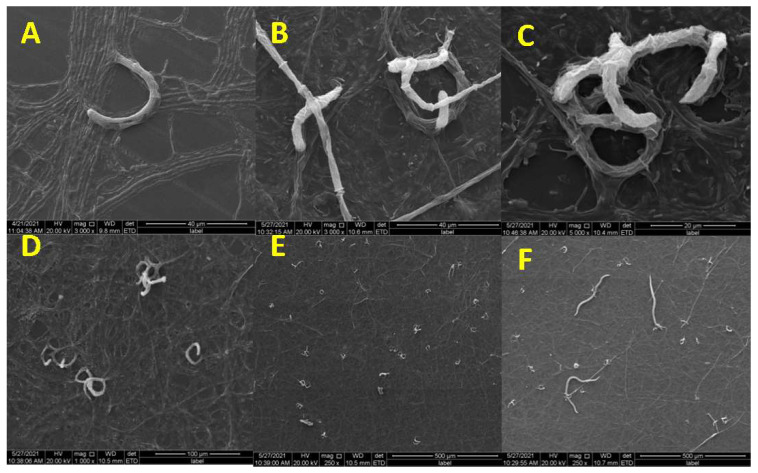
Trap (adhesive three-dimensional network) formation of *D. flagrans*. (**A**) The loop was formed; (**B**) The connection of hyphal and loop; (**C**) The formation of a three-dimensional capture structure; (**D**) and (**E**): Many adhesive networks; (**F**) The captured nematodes.

NTF *D. flagrans* produces three-dimensional adhesive networks that trap nematodes. The trap formation and predatory process of *D. flagrans* against *M. incognita* were recorded using SEM. After nematodes were added to *D. flagrans* for 12 h, a simple loop formed from vegetative hypha, or at least two-thirds of a loop was formed (Figure 1A). New hyphal connections from the simple loop, or from another branch of the vegetative hyphae (Figure 1B), were observed. This resulted in the formation of a three-dimensional capture structure (Figure 1C). At the same time, many adhesive networks were observed (Figure 1D,E), and several nematodes were caught by the traps (Figure 1F).

**Figure 2:**
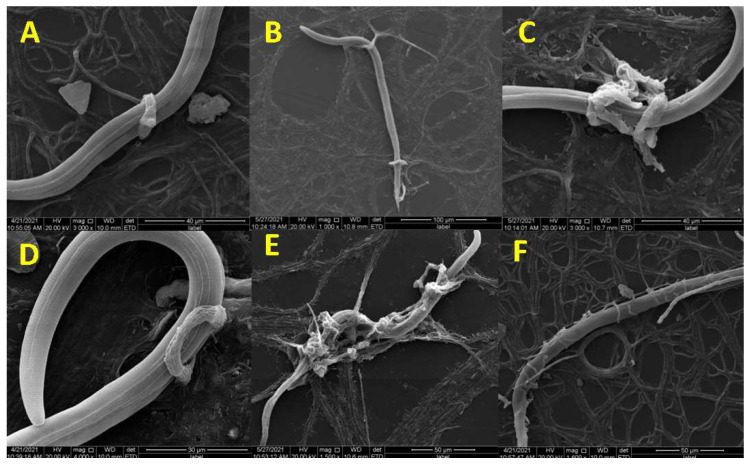
The pathogenicity process of *D. flagrans* against *M. incognita*. (**A**) The nematode was trapped by one loop; (**B**) The nematode was trapped at different portions; (**C**) The expanded three-dimensional network; (**D**) The nematode was adhered at the surface of the trap; (**E**) The dead nematode; (**F**) The nematode was twined by mycelia.

The nematodes were captured by the loops 12 h after inoculation of the fungus with *M. incognita*. At 24 h, many nematodes were trapped by at least one loop in a variety of locations, including their cephalic portion, body, or tail (Figure 2A). Different portions were even trapped at the same time (Figure 2B). With the advance of the predatory process, an increasing number of *M. incognitas* were captured and killed by traps, and an increase in the size of the three-dimensional network was observed (Figure 2C). In addition, we found that some nematodes adhered to the surface of the capture structure (Figure 2D), indicating that adhesive substances played an important role in the pathogenicity process. After 48 h, most nematodes died and were destroyed during the interaction process with *D. flagrans* (Figure 2E). This is similar to the predatory process of *D. flagrans* against *Ancylostoma* spp. of nematodes [27]. In addition, some nematodes twined by mycelia were observed in the pathogenicity process (Figure 2F). 

### 3.2. Nematicidal Activity of D. flagrans’ Broth VOCs against M. incognita

**Figure 3:**
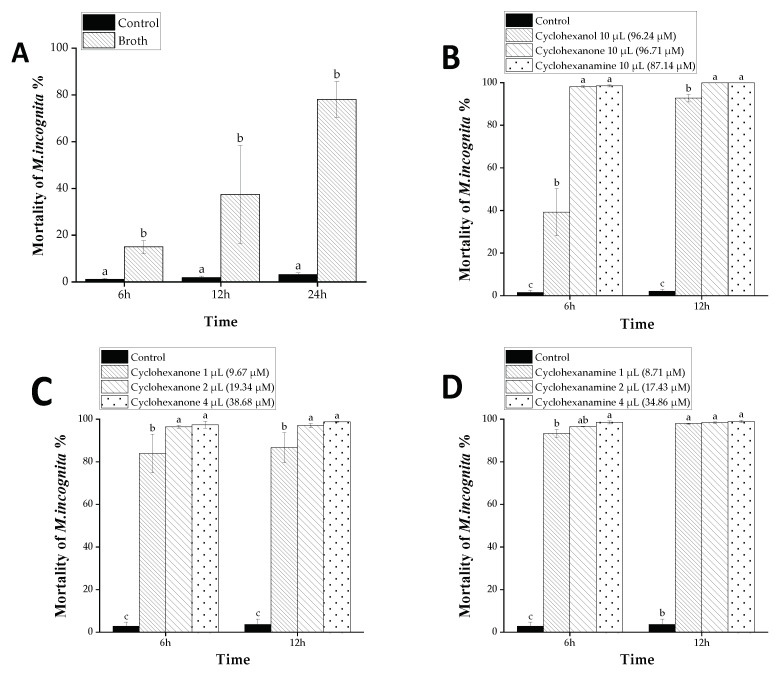
Nematicidal activity of VOCs produced by *D. flagrans* against *M. incognita*. Values with the same lowercase letters in the figure have no difference when *p* < 0.05; bars indicate the standard error of the means (*n* = 3). (**A**) The mortality of nematodes treated with broth and control; (**B**) The mortality of nematodes treated with cyclohexanol, cyclohexanone and cyclohexanamine; (**C**) The mortality of nematodes treated with different doses of cyclohexanone; (**D**) The mortality of nematodes treated with different doses of cyclohexanamine.

*D. flagrans* was cultured in YPD medium for 14 days. The nematicidal activity of VOCs, produced by *D. flagrans*, was assayed in a 96-well plate. It showed strong activity against *M. incognita* (Figure 3). It caused 78.10% mortality at 24 h, which was much higher than that of the medium control (Figure 3A). The results revealed that *D. flagrans* could produce VOCs to kill *M. incognita*. 

### 3.3. Identification of the VOCs Produced by D. flagrans

According to the GC-MS results, 52 VOCs were identified in the broth extracts of *D. flagrans* (Supplementary File). Eleven main volatile compounds (cyclohexanol, cyclohexanone, cyclohexanamine, heptane, 2-pyrrolidinone, benzeneethanol, tetradecane, 1-dodecanol, cyclododecane, 9-octadecenoic acid and tetracosane) were selected mainly based on area percentage. These 11 metabolites were considered as the active candidates, and their nematicidal effects were assayed, using commercial compounds. 

### 3.4. Nematicidal Activity of VOCs against M. incognita 

The activity of VOCs against *M. incognita* was tested in 96-well plates. At a dose of 10 µL, three of the 11 tested candidates (cyclohexanol, cyclohexanone and cyclohexanamine) exhibited strong nematicidal activity against *M. incognita* (Figure 3B). Cyclohexanone and cyclohexanamine caused 100% mortality of *M. incognita* at 12 h. However, the other eight metabolites showed no obvious activity at 10 µL (Supplementary File). Subsequently, the activity of cyclohexanone and cyclohexanamine against *M. incognita* was further tested at lower doses, respectively. The results showed that the activity of cyclohexanone and cyclohexanamine was still strong at a dose of 9.67 and 8.71 µM, respectively, which led to 86.65% and 97.93% death rate, respectively, at 12 h (Figure 3C,D). 

### 3.5. Inhibition of Egg Hatching by VOCs

The inhibition of egg hatching by VOCs from the broth was tested. The number of nematodes was much lower within the presence of VOCs, than that in the medium control. The average number of hatched juveniles per egg mass after 3 days of treatment with VOCs from the broth was 15.35; whereas the average number under the control treatment was 99.72 (Table 1).

The three VOCs, cyclohexanamine, cyclohexanone, and cyclohexanol, were also tested for their inhibitory activity against egg hatching. Cyclohexanamine exhibited the highest inhibitory activity. The average number of hatched worms per egg mass after 3 days was 8.44, when treated with 26.14 µM cyclohexanamine (Table 1). Another compound, cyclohexanone, also showed strong inhibitory activity. With 96.71 µM and 48.36 µM cyclohexanone, the average numbers of hatched juveniles per egg mass after 3 days were 3.14 and 10.14, respectively. Among the three VOCs, cyclohexanol showed the weakest inhibitory activity on egg hatching, and the number of hatched juveniles was 30.69 after treatment with 96.24 µM of the compound for 3 days. 

## 4. Discussion

To date, many effective chemicals used for controlling the root-knot nematodes, *Meloidogyne* spp., are highly toxic and are gradually being forbidden. Therefore, less toxic nematicidal agents need to be developed. The lack of safer and more effective nematicides has accelerated the search for more natural products. In the last few years, much effort has been focused on the search for natural nematicidal VOCs, because their nematocidal toxicity is considered a possible alternative [28]. The VOCs can affect nematodes by direct contact or fumigation [24]. As a typical NTF, *D. flagrans* is famous for producing chlamydospores and three-dimensional adhesive networks that capture nematodes. To date, this is the most potential candidate for commercial application. Along with this, the species has been used to control parasite nematodes in animals [10].

SEM was used to observe the predatory process of *D. flagrans* against *M. incognita*. The species infects *M. incognita* by trapping the nematode with adhesive networks. The infection process observation of the species against *Haemonchus contortus* [29], *Strongylus equinus* [30], *Caenorhabditis elegans* [31] and *Ancylostoma* spp. [27] has been previously reported. This indicates that the species can capture different types of nematodes.

In this study, 52 VOCs were detected from the NTF *D. flagrans* using GC-MS. We then examined the activity of the 11 abundant VOCs against the root-knot nematode *M. incognita*. The results showed cyclohexanamine, cyclohexanone, and cyclohexanol were active against both juveniles and eggs. To the best of our knowledge, these molecules have never been tested in bioassays against the root-knot nematode *M. incognita*. Among them, cyclohexanamine exhibited the highest nematicidal activity, which indicates that NTFs could become an important source of natural nematicides. To date, thousands of VOCs produced by microorganisms have been identified that could result in new nematicides which may lead to new products development [16]. However, laboratory studies are not sufficient as they do not represent the natural environment [32]. Soil-based environmental experiments are required to demonstrate efficacy in situ.

In our study, *D. flagrans* efficiently trapped the plant’s parasitic nematode *M. incognita* and also produced VOCs to kill nematodes and inhibit egg hatching of *M. incognita*. VOC metabolites seem to have a synergistic effect regarding the pathogenic process of NTF *D. flagrans* against *M. incognita*. It has been shown that *D. flagrans* could reduce juveniles of *M. javanica* in soil [13]. These results indicate that *D. flagrans* has the potential to biocontrol plant root-knot nematodes. The biocontrol effects of a mixture of VOCs or a single VOC produced by the fungus, or the strain itself need to be tested further in soil experiments.

## Figures and Tables

**Table 1:** Effects of three VOCs and culture broth on egg hatching of *M. incognita*.

	Hatched Worms per Egg Mass ± SD		
	1 Day	2 Days	3 Days
Cyclohexanol 96.24 µM	29.13 ± 5.95 b	30.41 ± 4.87 b	30.69 ± 4.59 b
Cyclohexanone 96.71 µM	2.25 ± 0.82 d	3.14 ± 0.33 d	3.14 ± 0.33 d
Cyclohexanone 48.36 µM	10.01 ± 1.21 cd	10.14 ± 1.23 cd	10.14 ± 1.23 c
Cyclohexanamine 26.14 µM	7.00 ± 1.21 cd	8.18 ± 0.64 cd	8.44 ± 0.69 cd
Culture broth 200 µL	14.13 ± 4.02 c	14.92 ± 4.01 c	15.35 ± 4.24 c
control	83.28 ± 2.76 a	93.50 ± 3.05 a	99.72 ± 2.71 a

The number represents the means of the replicates ± SD. The same lowercase letters indicate that there is no significant difference between treatments (*p* < 0.05).

## Data Availability

Not applicable.

## Supplementary Materials

The following are available online at https://www.mdpi.com/article/10.3390/microorganisms9112268/s1. Supplementary File: The GC-MS data and nematicidal activity of metabolites.
