# Supplementary material for: Pathogenicity and Volatile Nematicidal Metabolites from Duddingtonia flagrans against Meloidogyne incognita
Source: Microorganisms. 2021 Oct 31;9(11):2268. doi: 10.3390/microorganisms9112268 (PMC8624258; doi:10.3390/microorganisms9112268)
Supplement: Supplementary file 1 [file microorganisms-09-02268-s001.zip › microorganisms-1420844-supplementary.pdf]

The GC-MS results of *Duddingtonia flagrans*

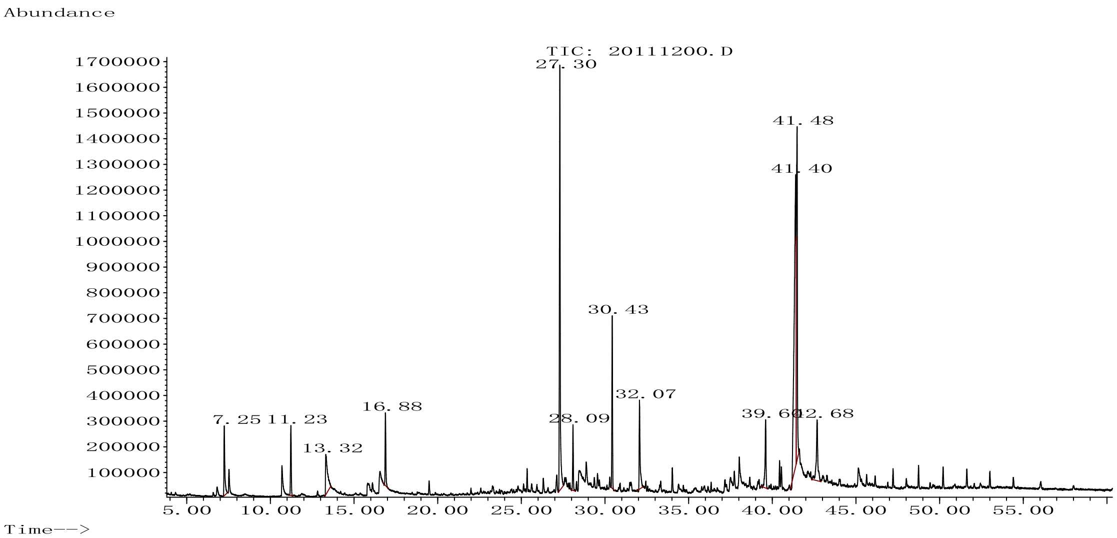

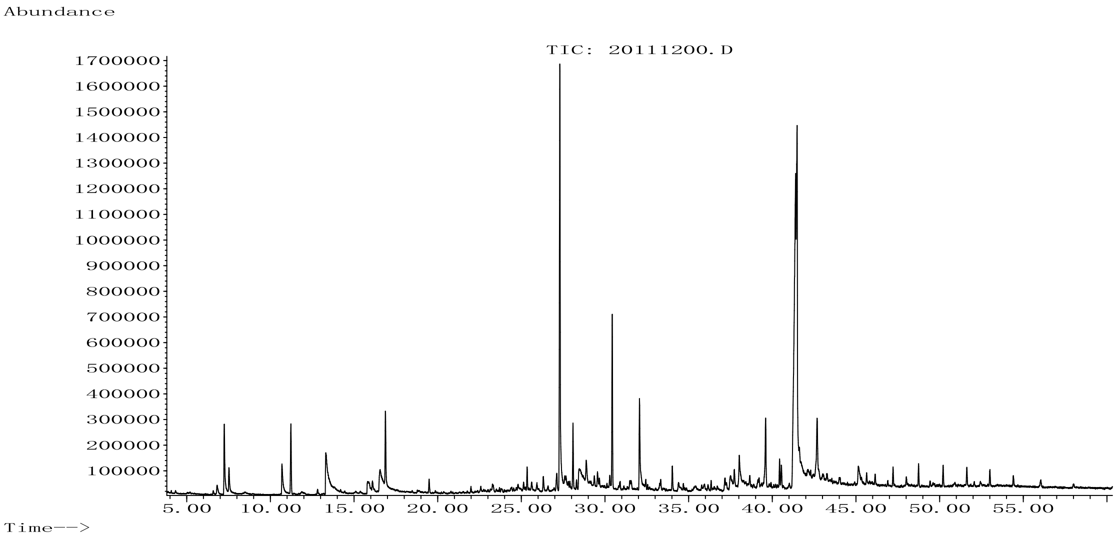

Area Percent Report

Data File : C:\MSDCHEM\1\DATA\20111200.D Vial: 1
Acq On : 12 Nov 2020 16:38 Operator:
Sample : MAF-GM Inst : GCMS
Misc : Multiplr: 1.00
Sample Amount: 0.00
MS Integration Params: autoint1.e

Method : C:\MSDCHEM\1\METHODS\D-DDVP.M (Chemstation Integrator)
Title :

Signal : TIC

peak #	R.T. min	first scan	max scan	last scan	PK TY	peak height	corr. area	corr. % max.	% of total
1	7.245	880	892	936	PV	274578	11504382	11.88%	2.052%
2	7.528	936	945	1026	VB 5	103703	5360091	5.54%	0.956%
3	10.696	1488	1538	1619	PV 2	117921	7900252	8.16%	1.409%
4	11.230	1619	1638	1659	VV	275514	8845405	9.14%	1.578%
5	13.319	2020	2029	2119	VV 3	160928	20600532	21.28%	3.675%
6	13.827	2119	2124	2183	VV 3	26565	3457214	3.57%	0.617%
7	15.878	2504	2508	2541	VV 10	43051	3308607	3.42%	0.590%
8	16.102	2541	2550	2596	VV 6	45273	2796148	2.89%	0.499%
9	16.556	2612	2635	2682	PV 4	83282	10367834	10.71%	1.849%
10	16.877	2682	2695	2764	VV 2	305142	12667376	13.08%	2.260%
11	24.805	4169	4179	4224	VV 2	30535	2778061	2.87%	0.496%
12	25.350	4269	4281	4294	VV 3	96247	3042172	3.14%	0.543%
13	26.317	4437	4462	4494	PV 3	56923	2556214	2.64%	0.456%
14	27.118	4597	4612	4630	VV 8	69718	3060900	3.16%	0.546%
15	27.305	4630	4647	4691	VV 2	1661307	59122732	61.06%	10.547%
16	27.663	4707	4714	4735	VV 8	56372	3484371	3.60%	0.622%
17	28.090	4780	4794	4823	VV	255720	7742529	8.00%	1.381%
18	28.470	4849	4865	4901	VV 6	84219	10969189	11.33%	1.957%
19	28.683	4901	4905	4927	VV 6	57495	4056289	4.19%	0.724%
20	28.881	4927	4942	4971	VV 7	113740	8382606	8.66%	1.495%
21	29.362	5011	5032	5058	VV 4	54085	3599664	3.72%	0.642%
22	29.554	5058	5068	5081	VV 5	68082	2961899	3.06%	0.528%
23	30.291	5183	5206	5219	VV 9	51358	2527331	2.61%	0.451%
24	30.436	5219	5233	5260	VV	685237	18917805	19.54%	3.375%
25	32.065	5526	5538	5595	VV 3	351927	16858043	17.41%	3.007%
26	34.026	5890	5905	5956	VV 2	97625	3474236	3.59%	0.620%
27	34.400	5956	5975	6018	VV 9	34553	2950838	3.05%	0.526%
28	35.425	6134	6167	6207	VV 9	20720	2874163	2.97%	0.513%
29	37.178	6463	6495	6505	PV 6	51897	2649296	2.74%	0.473%
30	37.503	6533	6556	6583	VV 4	60439	5988987	6.19%	1.068%
31	37.728	6583	6598	6620	VV 9	83979	4921042	5.08%	0.878%
32	38.032	6637	6655	6698	VV 4	136392	11848258	12.24%	2.114%
33	38.657	6756	6772	6787	VV 8	59618	3435436	3.55%	0.613%
34	39.603	6911	6949	6978	VV 4	278898	14405728	14.88%	2.570%
35	40.436	7092	7105	7114	VV 5	119613	3725792	3.85%	0.665%
36	40.548	7114	7126	7139	VV 8	95699	3696097	3.82%	0.659%
37	41.398	7233	7285	7290	VV 8	1207526	96820174	100.00%	17.271%
38	41.478	7290	7300	7321	VV 7	1404363	67097490	69.30%	11.969%
39	41.617	7321	7326	7408	VV 7	163348	24569575	25.38%	4.383%
40	42.135	7408	7423	7445	VV 7	76958	7773995	8.03%	1.387%
41	42.295	7445	7453	7470	VV 10	72602	4491826	4.64%	0.801%
42	42.445	7470	7481	7496	VV 10	57210	4477079	4.62%	0.799%
43	42.680	7496	7525	7566	VV 4	273649	23004712	23.76%	4.104%
44	43.017	7566	7588	7609	VV 6	56829	6338107	6.55%	1.131%
45	43.273	7624	7636	7654	VV 6	56539	4085124	4.22%	0.729%

46	43.460	7654	7671	7682	VV 6	34927	2739017	2.83%	0.489%
47	45.148	7958	7987	8016	VV 10	81627	7842946	8.10%	1.399%
48	45.640	8068	8079	8099	VV 6	56980	2785183	2.88%	0.497%
49	46.147	8163	8174	8213	VV 5	50110	2899447	2.99%	0.517%
50	48.743	8643	8660	8680	VV 2	89296	2725935	2.82%	0.486%
51	50.213	8923	8935	8956	VV 4	84754	2741638	2.83%	0.489%
52	51.628	9164	9200	9222	VV 3	76498	3349732	3.46%	0.598%

Sum of corrected areas: 560579501

Information from Data File:

File: C:\MSDCHEM\1\DATA\20111200.D
Operator:
Date Acquired: 12 Nov 2020 16:38
Method File: DLK
Sample Name: MAF-GM
Misc Info:
Vial Number: 1

Search Libraries: C:\Database\wiley7n.l Minimum Quality: 0

Unknown Spectrum: Apex minus start of peak
Integration Events: Chemstation Integrator - autoint1.e

Pk#	RT	Area%	Library/ID	Ref#	CAS#	Qual
1	7.24	2.05	C:\Database\wiley7n.l			
			Cyclohexanol $$ Adronal $$ Adro...	8465	000108-93-0	95
			Cyclohexanol (CAS) $$ Anol $$ N...	8469	000108-93-0	95
			Cyclohexanol (CAS) $$ Anol $$ N...	8473	000108-93-0	94
2	7.53	0.96	C:\Database\wiley7n.l			
			Cyclohexanone $$ Anon $$ Anone ...	7095	000108-94-1	90
			Cyclohexanone	7094	000108-94-1	90
			Cyclohexanone	7093	000108-94-1	90
3	10.70	1.41	C:\Database\wiley7n.l			
			Cyclohexanamine, N,N-dimethyl- ...	23795	000098-94-2	91
			Cyclohexanamine, N,N-dimethyl-	23794	000098-94-2	83
			3-Piperidinone, 1-ethyl- $$ 1-E...	23649	043152-93-8	78
4	11.23	1.58	C:\Database\wiley7n.l			
			Heptane, 2,2,4,6,6-pentamethyl-	74449	013475-82-6	83
			Heptane, 2,2,4,6,6-pentamethyl-...	74451	013475-82-6	83
			Heptane, 2,2,4,6,6-pentamethyl-...	74452	013475-82-6	83
5	13.32	3.67	C:\Database\wiley7n.l			
			2-Pyrrolidinone, 1-methyl- (CAS...	7640	000872-50-4	90
			2-Pyrrolidinone, 1-methyl- $$ M...	7643	000872-50-4	80
			2-Pyrrolidinone, 1-methyl- (CAS...	7645	000872-50-4	78
6	13.83	0.62	C:\Database\wiley7n.l			

Acetoxy-(3-aminopropyl)butylborane 93915 000000-00-0 43
Cyclopropane carboxylic acid, 1... 8073 000000-00-0 38
4-Methyltetrahydropyran 8646 000000-00-0 30

7 15.88 0.59 C:\Database\wiley7n.l
3-azido-2-pyridone $$ 2(1H)-Pyr... 30629 022714-02-9 38
ethyl 2-methylbuta-2,3-dienoate 22521 005717-41-9 38
4-azido-5-tert-butyl-2-cyano-2-... 161642 074987-59-0 28

8 16.10 0.50 C:\Database\wiley7n.l
Nonanal 38507 000124-19-6 80
Nonanal (CAS) $$ n-Nonanal $$ n... 38508 000124-19-6 64
Dodecanal (CAS) $$ n-Dodecanal ... 93114 000112-54-9 35

9 16.56 1.85 C:\Database\wiley7n.l
Benzeneethanol (CAS) $$ Pheneth... 19944 000060-12-8 91
Phenylethyl Alcohol 19948 000060-12-8 91
Benzeneethanol (CAS) $$ Pheneth... 19951 000060-12-8 91

10 16.88 2.26 C:\Database\wiley7n.l
Octanoic acid, methyl ester (CA... 57752 000111-11-5 94
Octanoic acid, methyl ester (CA... 57750 000111-11-5 91
Octanoic acid, methyl ester (CA... 57748 000111-11-5 91

11 24.80 0.50 C:\Database\wiley7n.l
Benzaldehyde, 3-hydroxy-4-metho... 48685 000621-59-0 30
Benzaldehyde, 4-hydroxy-3-metho... 48696 000121-33-5 30
Vanillin 48699 000121-33-5 27

12 25.35 0.54 C:\Database\wiley7n.l
Tetradecane (CAS) $$ n-Tetradec... 113292 000629-59-4 97
Tetradecane 113289 000629-59-4 96
Tetradecane $$ n-Tetradecane $$... 113291 000629-59-4 95

13 26.32 0.46 C:\Database\wiley7n.l
Butanoic acid, hexyl ester (CAS... 76417 002639-63-6 64
Butanoic acid, hexyl ester (CAS... 76419 002639-63-6 56
Propanoic acid, 2-methyl-, hexy... 76451 002349-07-7 53

14 27.12 0.55 C:\Database\wiley7n.l
2,5-Cyclohexadiene-1,4-dione, 2... 144342 000719-22-2 92
2,5-Cyclohexadiene-1,4-dione, 2... 144346 000719-22-2 91
2,5-Cyclohexadiene-1,4-dione, 2... 144343 000719-22-2 91

15 27.31 10.55 C:\Database\wiley7n.l
1-Dodecanol (CAS) $$ n-Dodecano... 95935 000112-53-8 95
1-Dodecanol 95931 000112-53-8 95
1-Dodecanol (CAS) $$ n-Dodecano... 95929 000112-53-8 95

16 27.66 0.62 C:\Database\wiley7n.l
Butanoic acid, propyl ester (CA... 26467 000105-66-8 72
Butanoic acid, butyl ester (CAS... 40296 000109-21-7 56
Butanoic acid, propyl ester (CA... 26460 000105-66-8 56

17 28.09 1.38 C:\Database\wiley7n.l
Decane, 5,6-dipropyl- 154957 119209-20-0 86

bis(2-Ethylhexyl) ether 177063 000000-00-0 80
Nonane, 2,3-dimethyl- $$ 2,3-Di... 56263 002884-06-2 64

18 28.47 1.96 C:\Database\wiley7n.l
Acetamide, N-(2-phenylethyl)- $... 63352 000877-95-2 90
PHENYLETHYLAMINE ACET. $$ P.E.A... 63410 000000-00-0 90
Acetamide, N-(2-phenylethyl)- 63353 000877-95-2 83

19 28.68 0.72 C:\Database\wiley7n.l
1-(phenylvinyl)-1-cyclopentene 74528 135312-78-6 35
Pyrimidine, 4-methyl-2-phenyl- ... 74126 034771-48-7 27
1,1-Difluoro-2-methyl-2-vinyl-c... 17728 000000-00-0 10

20 28.88 1.50 C:\Database\wiley7n.l
2,3-Dimethyl-1,4-dioxa-spiro[4.... 112751 000000-00-0 53
4-METHOXY-6-PENTYL-5,6-DIHYDRO-... 112203 000000-00-0 49
3,5-Octanedione, 2,2,7-trimethyl- 92851 069725-37-7 47

21 29.36 0.64 C:\Database\wiley7n.l
3-AMINO-5-DIMETHYLAMINO-1,2,4-T... 23414 051108-33-9 53
4-NITRO-1-METHYLPYRAZOLE $$ 4-N... 23345 003994-50-1 52
1-Methyl-3-nitropyrazole 23337 054210-32-1 50

22 29.55 0.53 C:\Database\wiley7n.l
1H-Imidazole, 4-methyl-5-nitro- 23382 014003-66-8 53
3-Butenoic acid, 2,2-diethyl-3-... 55271 038477-06-4 53
1H-Imidazole, 1-methyl-5-nitro-... 23371 003034-42-2 52

23 30.29 0.45 C:\Database\wiley7n.l
Hexadecane $$ n-Cetane $$ n-Hex... 154910 000544-76-3 78
Hexadecane (CAS) $$ n-Hexadecan... 154903 000544-76-3 60
Hexadecane (CAS) $$ n-Hexadecan... 154916 000544-76-3 53

24 30.44 3.37 C:\Database\wiley7n.l
2-Ethylhexyl 2-ethylhexanoate $... 195503 007425-14-1 91
2-Ethylhexyl 2-ethylhexanoate $... 195502 007425-14-1 90
2-Ethylhexyl 2-ethylhexanoate 195505 007425-14-1 52

25 32.07 3.01 C:\Database\wiley7n.l
Cyclododecane 71563 000294-62-2 94
Cyclododecane 71568 000294-62-2 93
Cyclododecane 71566 000294-62-2 93

26 34.03 0.62 C:\Database\wiley7n.l
Silane, dimethyl-2-propenyl(tet... 263186 077774-33-5 64
N-{8-[(2-Methoxycarbonyl-acetyl... 314840 000000-00-0 40
3-Acridinol, 9-phenyl- 215035 109553-65-3 38

27 34.40 0.53 C:\Database\wiley7n.l
1,3-DIPHENYL-1,3,5,5-TETRAMETHY... 294715 000000-00-0 59
1-Methyl-3,6-diazahomoadamantan... 190827 000000-00-0 38
1,3-Diphenyl-4H-1,2,4-triazolin... 191067 005055-73-2 35

28 35.43 0.51 C:\Database\wiley7n.l
1,3-Dimethyl-melamine 51410 000000-00-0 27
Phenol, 3,5-dimethoxy-, acetate... 108701 023133-74-6 27

1,2,3-Trimethyl-5-(2-thia-n-hex... 177560 000000-00-0 22

29 37.18 0.47 C:\Database\wiley7n.l
vinylbital 150464 000000-00-0 35
N-METHYL-2-PROPYL-5-BUTYLPIPERI... 111130 000000-00-0 35
1,1'-Biphenyl (CAS) $$ Biphenyl... 53748 000092-52-4 30

30 37.50 1.07 C:\Database\wiley7n.l
p-(Methylthio)benzyl alcohol $$... 51750 003446-90-0 43
(+-)Boschnialactone $$ Cyclopen... 52265 016802-12-3 42
2,8-Diazaspiro(4,4)-nonane-1,9-... 51628 000000-00-0 37

31 37.73 0.88 C:\Database\wiley7n.l
Silane, (hexadecyloxy)trimethyl- 265324 006221-90-5 47
n-Tridecanoic acid, trimethylsi... 233386 000000-00-0 38
n-Pentadecanoic acid, trimethyl... 265153 000000-00-0 35

32 38.03 2.11 C:\Database\wiley7n.l
1,2-Benzenedicarboxylic acid, b... 223230 017851-53-5 38
1,2-Benzenedicarboxylic acid, m... 167331 024539-56-8 38
1,2-Benzenedicarboxylic acid, b... 254298 000084-64-0 38

33 38.66 0.61 C:\Database\wiley7n.l
Eicosane (CAS) $$ n-Eicosane 228990 000112-95-8 83
Eicosane $$ n-Eicosane 228983 000112-95-8 83
Eicosane (CAS) $$ n-Eicosane 228982 000112-95-8 70

34 39.60 2.57 C:\Database\wiley7n.l
Hexadecanoic acid, trimethylsil... 279149 055520-89-3 83
Hexadecanoic acid, trimethylsil... 279147 055520-89-3 38
Hexadecanoic acid, trimethylsil... 279148 055520-89-3 35

35 40.44 0.66 C:\Database\wiley7n.l
9,12-Octadecadienoic acid, meth... 243102 002566-97-4 99
10,13-Octadecadienoic acid, met... 243108 056554-62-2 97
9,12-Octadecadienoic acid, meth... 243170 002462-85-3 95

36 40.55 0.66 C:\Database\wiley7n.l
11-Octadecenoic acid, methyl ester 245515 052380-33-3 98
14-Octadecenoic acid, methyl ester 245519 056554-48-4 96
9-Octadecenoic acid (Z)-, methy... 245476 000112-62-9 95

37 41.40 17.27 C:\Database\wiley7n.l
9,12-Octadecadienoic acid (Z,Z)... 226103 000060-33-3 99
9,12-Octadecadienoic acid (Z,Z)... 226101 000060-33-3 97
9,12-Octadecadienoic acid (Z,Z)- 226099 000060-33-3 95

38 41.48 11.97 C:\Database\wiley7n.l
9-Octadecenoic acid, (E)- 228774 000112-79-8 94
HEPTADECENE-(8)-CARBONIC ACID-(1) 228686 000000-00-0 93
9-Octadecenoic acid (Z)- (CAS) ... 228694 000112-80-1 91

39 41.62 4.38 C:\Database\wiley7n.l
2-benzoyl-6,7-dimethoxy-4-methy... 277921 000000-00-0 86
2,4-Diphenyl-3-(methylthio)-5-n... 277863 072525-35-0 78
1-Dimethyl(chloromethyl)silylox... 317916 000000-00-0 72

Pk#	RT	Area%	Library/ID	Ref#	CAS#	Qual
40	42.14	1.39	C:\Database\wiley7n.l			
			Hexadecanamide	193941	000629-54-9	83
			9-Octadecenamide, (Z)- (CAS) $$...	227140	000301-02-0	80
			Octadecanamide $$ Stearamide $$...	229847	000124-26-5	80
41	42.30	0.80	C:\Database\wiley7n.l			
			Octadecane, 2,6,10,14-tetrameth...	261320	054964-82-8	70
			Docosane (CAS) $$ n-Docosane $$...	261309	000629-97-0	70
			(2S,3R)-3-dimethyl-t-butylsilox...	181197	097848-37-8	64
42	42.44	0.80	C:\Database\wiley7n.l			
			t-Butyl (3S)-2-(5,6-di-O-acetyl...	357865	087436-58-6	17
			2-Acetamido-8-hydroxy-10,10-dim...	243927	000000-00-0	10
			9,10-Dihydro-9,10-([1',7']-tric...	244140	078365-74-9	9
43	42.68	4.10	C:\Database\wiley7n.l			
			Oleic acid, trimethylsilyl ester	301854	021556-26-3	97
			11-TRANS-OCTADECENOIC ACID 1TMS	301861	000000-00-0	96
			Oleic acid, trimethylsilyl este...	301853	000000-00-0	95
44	43.02	1.13	C:\Database\wiley7n.l			
			1-Naphthalenamine, N-phenyl-	143056	000090-30-2	76
			Nonox A $$ 1-Naphthalenamine, N...	143057	000090-30-2	60
			2-Naphthalenamine, N-phenyl- (C...	143068	000135-88-6	59
45	43.27	0.73	C:\Database\wiley7n.l			
			1,1'-Biphenyl, 3-nitro- $$ Biph...	113852	002113-58-8	30
			1,1'-Biphenyl, 3-nitro- $$ Biph...	113854	002113-58-8	30
			Acenaphthylene, 1,2-dihydro-5-n...	113869	000602-87-9	27
46	43.46	0.49	C:\Database\wiley7n.l			
			Tributyl acetylcitrate	333403	000077-90-7	43
			Tributyl acetylcitrate $$ 1,2,3...	333402	000077-90-7	37
			Ethanone, 1-(4-bromophenyl)-2,2...	188728	016184-89-7	30
47	45.15	1.40	C:\Database\wiley7n.l			
			9-Octadecenamide, (Z)- $$ Adoge...	227139	000301-02-0	86
			9-Octadecenamide, (Z)-	227138	000301-02-0	83
			Erucylamide $$ 13-Docosenamide,...	287552	000112-84-5	64
48	45.64	0.50	C:\Database\wiley7n.l			
			Tetracosane	288783	000646-31-1	91
			Tetracosane (CAS) $$ n-Tetracosane	288788	000646-31-1	91
			Octadecane	193176	000593-45-3	89
49	46.15	0.52	C:\Database\wiley7n.l			
			Methyl n-amyl disulfide $$ Disu...	45811	072437-68-4	40
			1-Methoxyphospholane sulfide	45662	078870-70-9	37
			3-Methyl-1,2-thiazolo[4,5-b]pyr...	45916	000000-00-0	25
50	48.74	0.49	C:\Database\wiley7n.l			
			Tetracosane (CAS) $$ n-Tetracosane	288787	000646-31-1	97
			Tetracosane	288783	000646-31-1	95
			Hexacosane	311168	000630-01-3	94

51   50.21   0.49 C:\Database\wiley7n.l
Heptacosane   320678 000593-49-7 94
Heptacosane $$ n-Heptacosane   320679 000593-49-7 93
9-methylnonadecane   229008 000000-00-0 93

52   51.63   0.60 C:\Database\wiley7n.l
Octacosane (CAS) $$ n-Octacosane   329269 000630-02-4 98
Octadecane (CAS) $$ n-Octadecan... 193184 000593-45-3 89
Docosane   261314 000629-97-0 89

Tue Feb 02 19:05:52 2021

The mortality of *M. incognita* treated with other eight metabolites (10 μL).

	Mortality of *M. incognita* %		
	**6 h**	**12 h**	**24 h**
Heptane	1.27 %	1.80 %	3.76 %
2-Pyrrolidinone	1.10 %	1.54 %	1.80 %
Benzeneethanol	1.04 %	1.55 %	2.48 %
Tetradecane	1.57 %	2.06 %	3.50 %
1-Dodecanol	1.22 %	1.53 %	2.40 %
Cyclododecane	0.86 %	1.47 %	2.23 %
9-Octadecenoic acid	1.12 %	1.45 %	2.70 %
Tetracosane	1.29 %	1.44 %	4.03 %
control	1.55 %	2.12 %	2.90 %
